# A Rare Case of Tumoral Calcinosis in a Chronic Hemodialysis Patient

**DOI:** 10.7759/cureus.68272

**Published:** 2024-08-31

**Authors:** Achraf Tebbaa El Hassali, Mohammed Barrached, Adnane Lachkar, Najib Abdeljaouad, Hicham Yacoubi

**Affiliations:** 1 Orthopedics and Traumatology, Faculty of Medicine and Pharmacy of Oujda, Mohammed VI University Hospital, Mohamed I University, Oujda, MAR

**Keywords:** surgery, chronic, renal failure, hemodialysis, tumoral, calcinosis

## Abstract

Tumor calcinosis is a rare condition. It is characterized by the presence of calcified masses in the juxta-articular regions without joint involvement. It particularly affects young adults and adolescents. Its exact pathogenesis remains poorly defined. The diagnosis is suspected clinically and radiologically but confirmed by histological examination. The treatment is mainly surgical, and the prognosis is often good. We report the original case of a chronic hemodialysis patient presenting with tumoral calcinosis by discussing our diagnostic and therapeutic approach according to data from the recent scientific literature.

## Introduction

Tumoral calcinosis, also known as Teutschlaender lipo-calcinogranulomatosis, is a very rare condition. It manifests itself by peri-articular or soft tissue-calcified deposits that can mimic tumor masses. It particularly affects adolescents and young adults of black skin with familial forms. Its exact etiopathogenesis remains poorly understood. The idiopathic form is probably linked to mutations in fibroblast growth factor 23 and GalNAc transferase 3 [1-3].

Several hypotheses have been put forward to explain the pathophysiology of these deposits involving several factors: a genetic predisposition, phospho-calcium metabolism disorders, chronic renal failure, and the role of even minimal trauma [4].

We report the case of a chronic hemodialysis patient presenting with tumoral calcinosis, discussing our diagnostic and therapeutic approach according to data from recent scientific literature.

## Case presentation

We report the case of a 49-year-old Moroccan male patient, with a clear phototype, and a trader by profession. His past medical history consisted of type II diabetes for eight years on metformin 1 g/day, high blood pressure on amlodipine 10 mg/day, and chronic renal failure due to undetermined nephropathy for three years on hemodialysis at a rate of three sessions per week. The patient had no pathological family history.

The patient consulted for a mass in the left gluteal region that appeared gradually two years previously. This mass was associated with other masses at the right shoulder, right elbow, and left wrist, all appearing successively after the gluteal mass. The latter had increased rapidly in size over the previous two months, making it difficult to walk and carry out daily activities, unlike the other non-progressive ones. On clinical examination, the patient was in good general condition, hemodynamically and respiratory stable (blood pressure of 140/70 mmHg, heart rate of 86 beats per minute, respiratory rate of 24 cycles per minute), and he was afebrile at 37°C.

The osteoarticular examination identified a swelling 30 cm long and 15 cm wide, of soft consistency, next to the trochanteric mass, not painful spontaneously or on palpation. This swelling was firm, hard, adherent to the deep planes, and without signs of inflammation nearby. The examination of the hip and knee was without abnormalities with preserved joint range of motion and a normal vascular-nervous examination.

**Figure 1:**
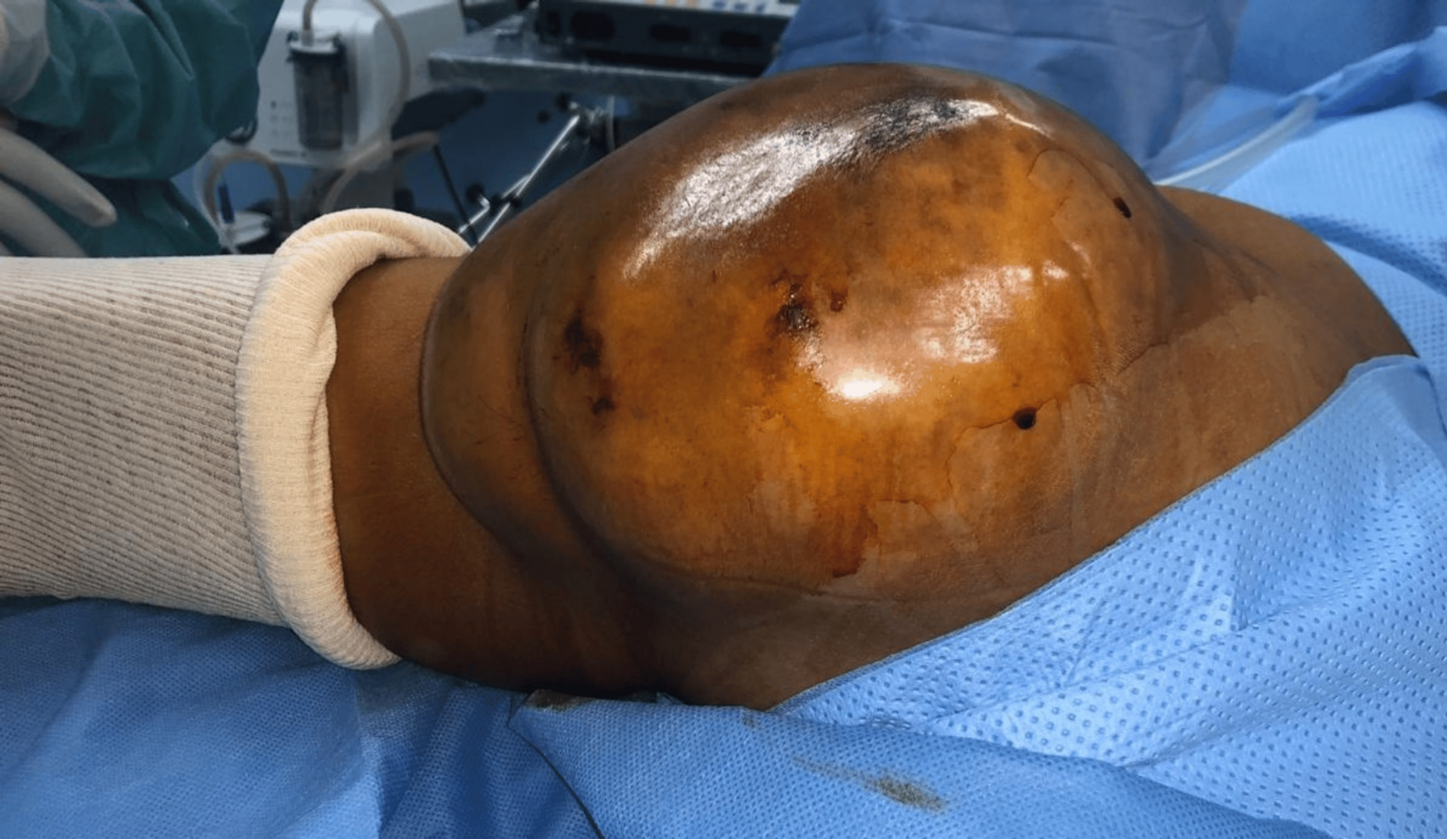
Swelling in the left gluteal region

**Figure 2:**
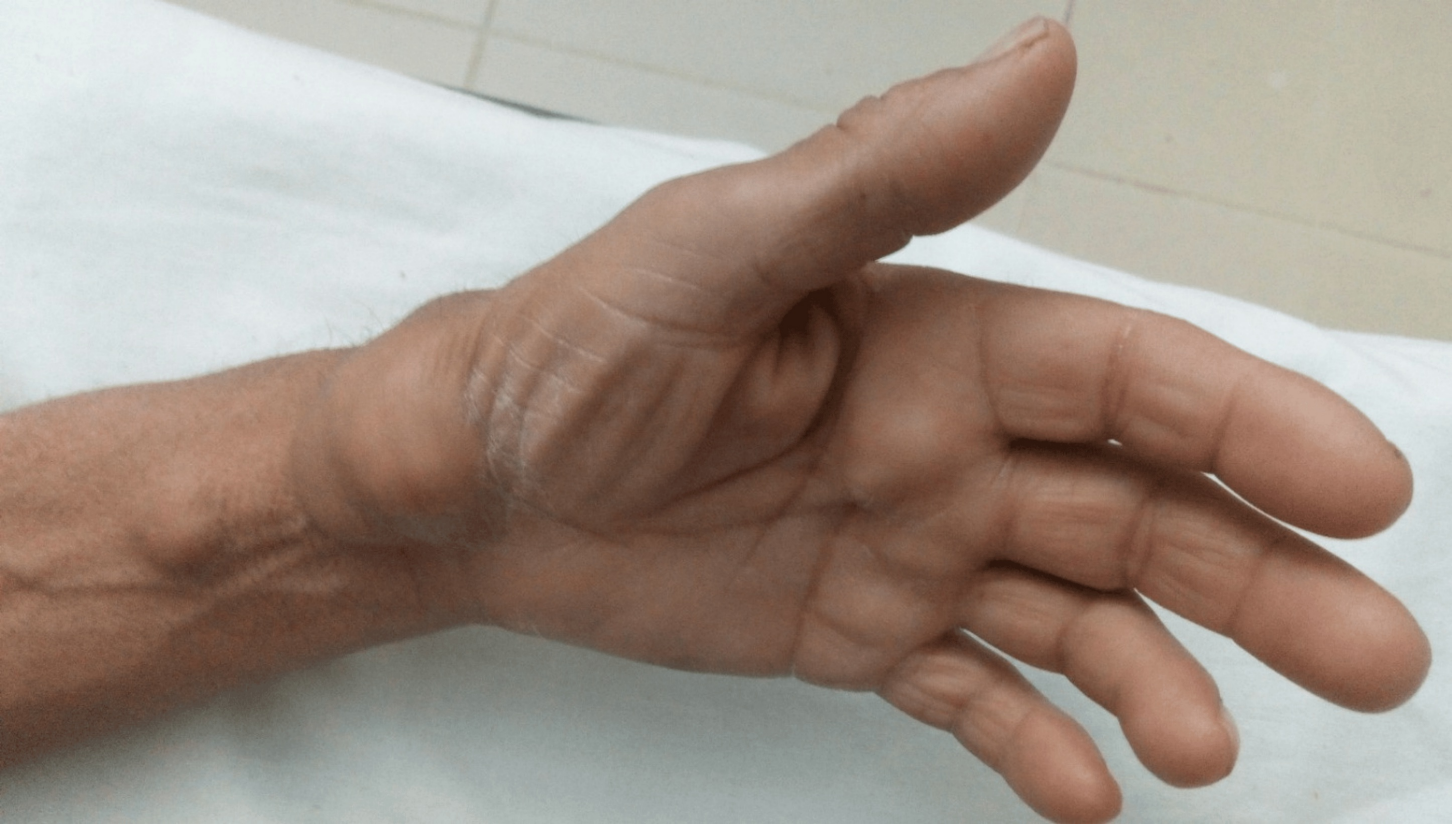
Left wrist swelling

**Figure 3:**
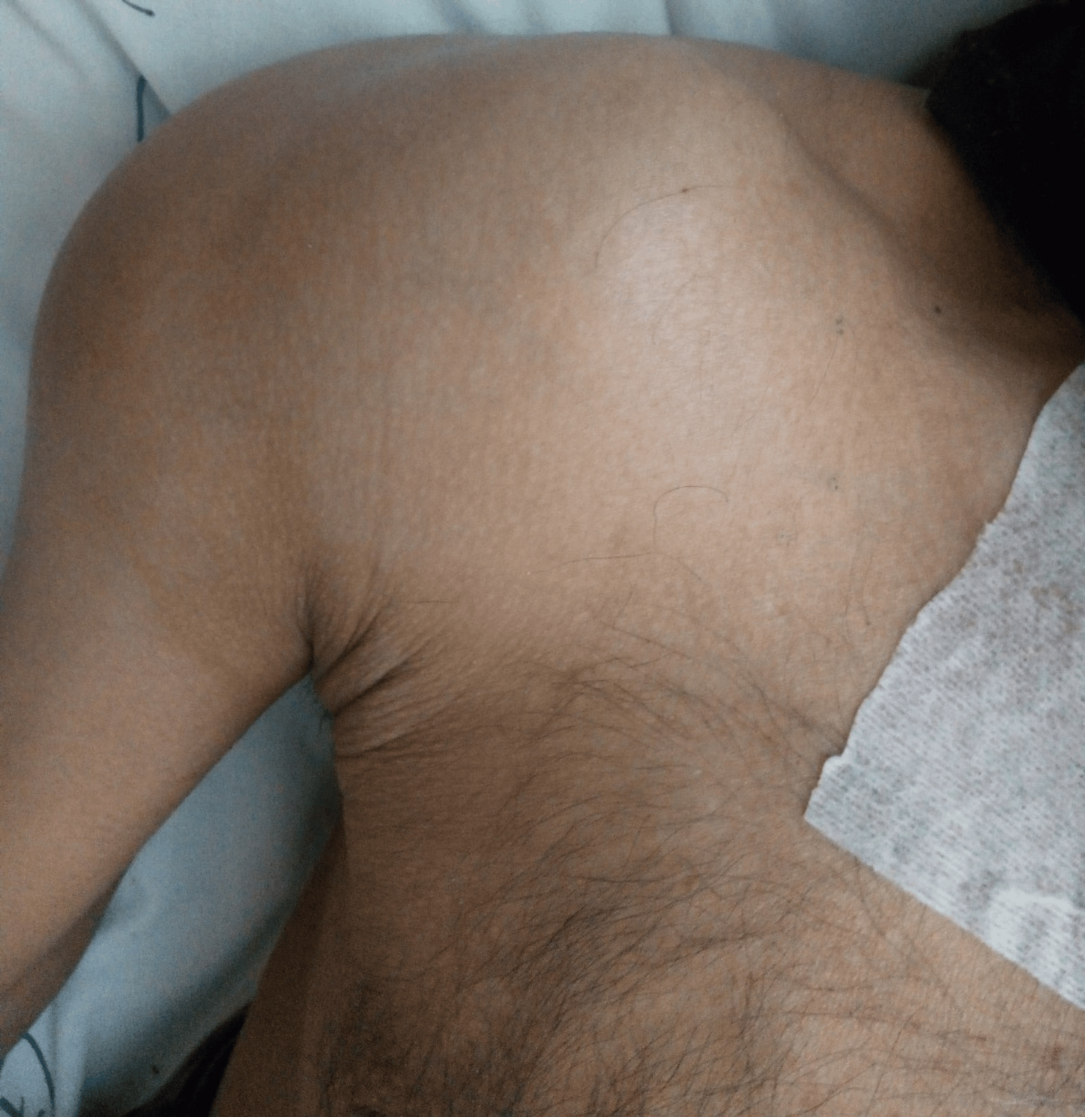
Swelling in the right shoulder

**Figure 4:**
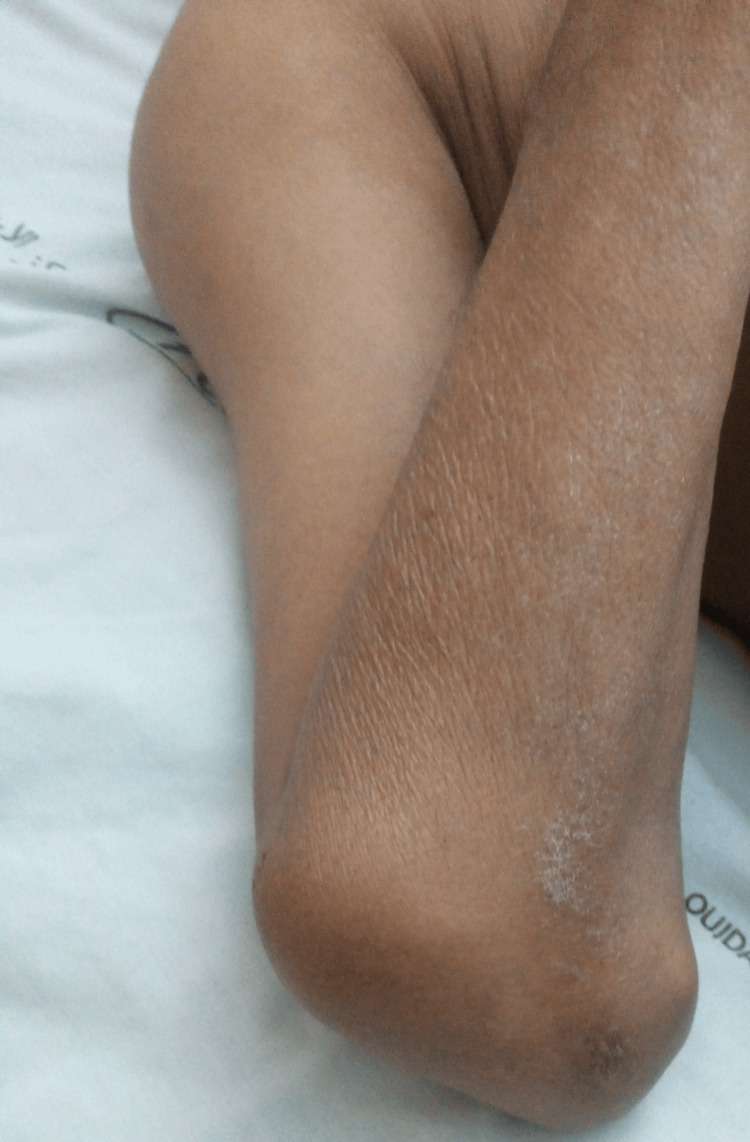
Swelling in the right elbow

The patient also presented with a swelling of 10 cm in length and width at the level of the right shoulder and 5 cm in length and width at the level of the right elbow, as well as the left wrist with the same characteristics of the gluteal mass initially described and with normal mobility and without vascular-nervous damage. The clinical appearance is presented in Figures 1-4.

The patient underwent a complete biological assessment, and the results are presented in Table 1.

**Table 1:** Results of the biological assessment CRP: C-reactive protein; PTH: Parathyroid hormone; TSH: thyroid-stimulating hormone

Setting	Patient value	Normal values
PTH	3995 pg/mL	15-65 pg/mL
Uric acid	65.20 mg/L	35-72 mg/L
Albumin	32 g/L	35-50 g/L
Calcium	92 mg/L	84-102 mg/L
Chlorine	95 mEq/L	98-107 mEq/L
Phosphorus	80 mg/L	23-47 mg/L
Potassium	5 mEq/L	3.5-5.1 mEq/L
Sodium	138 mEq/L	135-145 mEq/L
Glucose	2.43 g/L	0.70-1.10 g/L
Creatinine	93.01 mg/L	7.2-12.5 mg/L
Urea	1.02 g/L	0.15–0.39 g/L
CRP	121.40 mg/L	< 5 mg/L
Total proteins	57 g/L	64-83 g/L
TSH	1.76 μIU/mL	0.35–4.94 μIU/mL
Hemoglobin	14 gd/L	12–16 gd/L
Platelets	250,000/μL	150,000–400,000/μL
White blood cells	8000/μL	4000–10,000/μL

**Figure 5:**
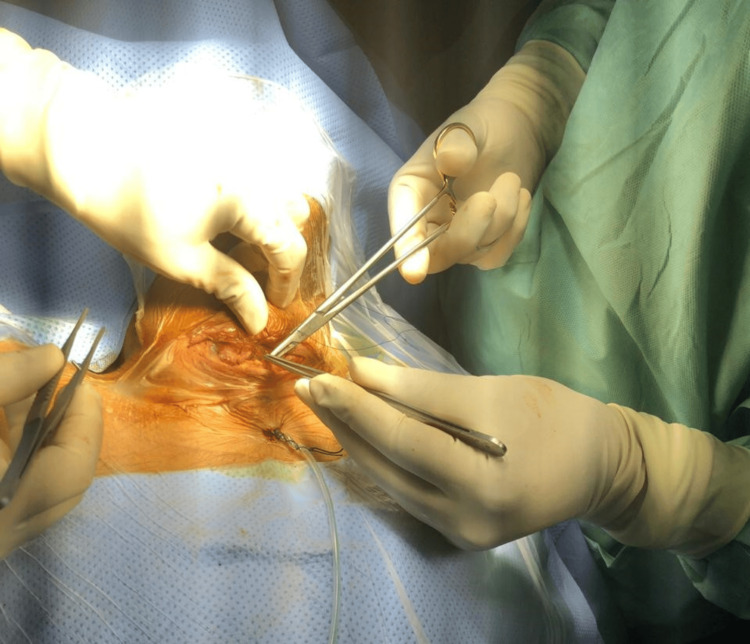
Operative image of parathyroidectomy

**Figure 6:**
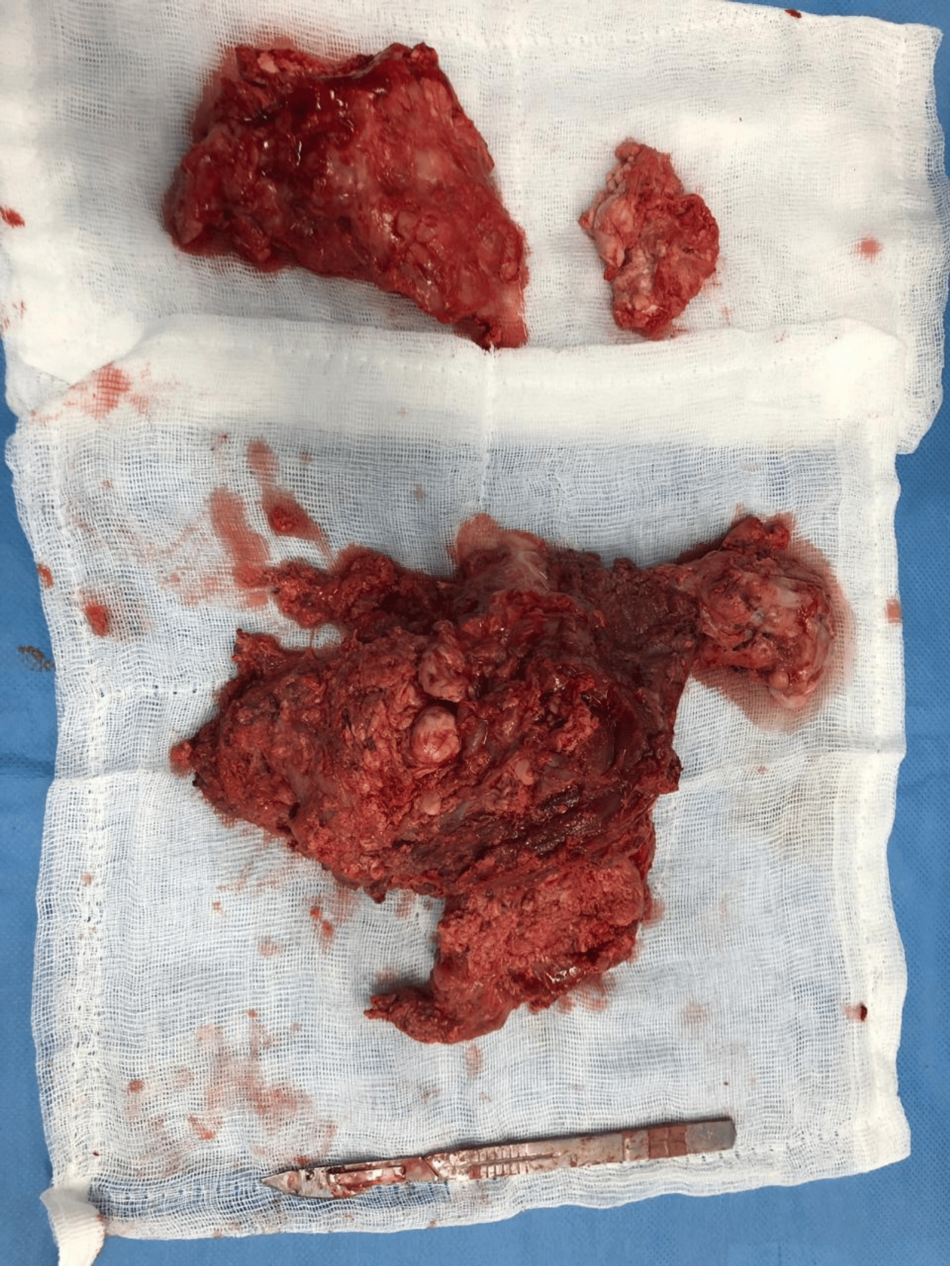
Surgical specimen for tumoral calcinosis of the hip

The patient received a standard radiograph of all affected areas (pelvis, elbow, shoulder), showing rounded, well-defined peri-articular calcifications. The radiological assessment was completed by a computed tomography (CT) of the pelvis showing a large, well-defined extra-osseous and extra-articular calcified mass, with heterogeneous content suggestive of tumoral calcinosis or a calcified tumor. The patient also benefited from a cervical ultrasound showing retrothyroid nodules, hypo-echoic probably related to parathyroid nodules. The patient benefited from a subtotal parathyroidectomy with surgical excision of the calcinosis tumor of the hip, as presented in the images operating procedures (Figures 5-6).

The patient was put on analgesic treatment based on paracetamol 3 g/day for a day associated with additional medical treatment based on phosphate chelators and a hypocalcium, hypophosphoric diet. The postoperative course was simple without any complications. The histological examination confirmed that it was a calcinosis tumor without signs of malignancy at the level of the hip, as well as at the level of the parathyroid nodules. Follow-up of the patient for two years has not identified any recurrence with spontaneous regression of the other masses.

## Discussion

Tumoral calcinosis is a rare entity, first reported by Inclan et al. in 1943 [3,5]. It can be primarily due to enzymatic hyperphosphatemia or secondary to chronic renal failure or secondary hyperparathyroidism [6,7]. Contrary to the literature, the case of our patient is distinguished by two particularities: the clear phototype, the absence of familial cases, and the delayed age of onset.

The clinical presentation depends on the location, volume, and duration of development. Classically, patients report pain or peri-articular swelling, which motivates their medical consultation. Calcinosis manifests itself by multiple calcified deposits, mimicking tumor and peri-articular masses. Their appearance is often gradual and without pain or inflammatory signs. Their size and location vary greatly. Published clinical cases suggest locations at the hip, wrist, elbow, shoulder, and foot [8,9].

Classification of histological lesions of tumoral calcinosis was proposed by Slavin et al., distinguishing three stages [10,11]: the first stage with cellular lesions devoid of calcifications, the second stage with cellular cystic lesions and calcifications, and the third stage associated with calcified hypocellular lesions.

The diagnosis of tumoral calcinosis is suggested in the face of the classic radiological appearance in the form of small rounded, well-defined, peri-articular opacities, giving a “honeycomb” appearance. CT and magnetic resonance imaging allow a more detailed study of lesions and relationships with muscles and neighboring structures. The presence of a fluid level suggests an active phase of the disease [12].On the biological level, we often find isolated hyperphosphatemia sometimes associated with an increase in vitamin D. In certain cases, we can find hypercalcemia or an increase in alkaline phosphatase and parathyroid hormone (PTH) [13].

Tumoral calcinosis can cause diagnostic confusion with bone tumors, Burnett syndrome, hypervitaminosis D, dermatomyositis, hyperparathyroidism, and chronic renal failure. Biopsy sometimes allows these differential diagnoses to be eliminated [14]. Treatment consists mainly of surgical excision, particularly in cases of discomfort related to the volume or location of the mass. Cases of spontaneous regression have also been reported [14]. Surgery can be combined with medical treatment based on phosphate chelators associated with a diet low in phosphate and calcium in order to limit the progression of the lesions or avoid recurrence [14]. The prognosis for the disease remains good.

## Conclusions

Tumoral calcinosis is a rare and benign condition. It is characterized by a deposit of calcium material in periarticular soft tissues, taking a benign tumor form. It most often affects adolescents and young adults. The clinical presentation is variable and non-specific. The diagnosis can be suspected in radiology, but diagnostic confirmation is based on the histological study of the biopsy or the surgical specimen after resection.

Spontaneous regression is possible, but surgical resection allows symptomatic improvement in the majority of cases. The prognosis remains good despite a significant recurrence rate.
